# *Scutellaria baicalensis* and *Lonicera japonica*: An In-Depth Look at Herbal Interventions Against Oxidative Stress in Non-Ruminant Animals

**DOI:** 10.3390/vetsci12090816

**Published:** 2025-08-26

**Authors:** Vetriselvi Sampath, Yu Jin Baek, In Ho Kim

**Affiliations:** 1 Department of Animal Biotechnology, Dankook University, Cheonan 31116, Republic of Korea; suve2314@gmail.com (V.S.); ujin2097@gmail.com (Y.J.B.); 2 Smart Animal Bio Institute, Dankook University, Cheonan 31116, Republic of Korea

**Keywords:** oxidative stress, pigs, poultry, herbal additives

## Abstract

The use of antibiotics as growth promoters (AGPs) has been a common practice in animal production; however, concerns over microbial resistance have led organizations, such as the FAO, EU, and EFSA, to restrict or ban their use. This has prompted a growing interest in natural alternatives, particularly medicinal herbs, for enhancing animal performance and health. Among these, *Scutellaria baicalensis* (Chinese Skullcap) and *Lonicera japonica* (Japanese Honeysuckle) have gained attention for their high medicinal value in monogastric animal diets. These plants contain bioactive compounds, such as flavones (baicalin, baicalein, oroxylin A), iridoids (loganin), and saponins (including loniceroside hederagenin), which exhibit a range of biological activities, including antioxidative, anti-inflammatory, antibacterial, antiviral, and anti-stress effects. Notably, these herbal extracts are natural, safe, and unlikely to induce microbial resistance. Recent studies suggest that supplementation with *S. baicalensis* and *L. japonica* can improve livestock production performance by mitigating oxidative stress. This review aims to highlight the potential application of these plant-based additives in reducing oxidative damage and enhancing productivity in animal agriculture.

## Simple Summary

Oxidative stress, caused by an overload of free radicals, can harm cells and reduce the performance and product quality of animals. Monogastric animals are especially more sensitive to environmental and dietary stressors. Using herbal feed additives rich in natural antioxidants can help balance the redox state in the body, improving both health and production efficiency. *Scutellaria baicalensis*, a medicinal herb, contains active compounds like baicalin and baicalein. Similarly, *Lonicera japonica* contains phenolic acids, flavonoids, polysaccharides, and volatile oils. These compounds offer antiviral, antibacterial, antioxidant, anti-inflammatory, hypoglycemic, and lipid-lowering effects. When included in the diet of monogastric animals, such as pigs and poultry, these additives help to reduce stress and help control diseases caused by harmful microorganisms. Overall, their inclusion in animal feed can enhance growth performance, support immune function by reducing oxidative stress, and contribute to a more sustainable approach to livestock production.

## 1. Introduction

Animal-derived foods play a vital role in the global food supply by providing essential nutrients, such as protein, fat, minerals, vitamin A, B vitamins, and more. Therefore, enhancing the health and productivity of food animals is directly linked to improvements in human health. In recent decades, livestock have increasingly been exposed to a variety of environmental stressors, such as temperature fluctuations, poor ventilation, and inadequate lighting, as well as nutritional challenges like oxidized fats, mycotoxins, and heavy metals. Among all body systems, the intestinal tract stands at the forefront of these challenges. It serves not only as the primary site for nutrient digestion and absorption but also as a critical barrier between the external environment and the body’s internal systems [1]. The intestinal epithelium, located at this critical interface, renews rapidly and functions under a unique oxygen exchange mechanism, making it especially vulnerable to oxidative stress. Oxidative stress (OS) occurs when the production of reactive oxygen species (oxidants) exceeds the capacity of the body’s antioxidant defense systems. In the intestines, excessive oxidants can overwhelm this defense, leading to oxidative damage and triggering inflammatory responses. These disruptions can damage the intestinal barrier, impair its function, and contribute to diseases such as necrotizing enteritis, coccidiosis, and Newcastle disease [2,3,4], and also compromise intestinal epithelial homeostasis in food animals, including endotoxin exposure, weaning stress, and oxidative stress. To date, OS has become a major concern in livestock production as it compromises the integrity of the mucosal barrier and disturbs the microbial balance, thereby reducing the efficiency of digestion and nutrient absorption, leading to decreased overall performance and productivity [5].

Plant-derived feed additives typically include herbs, spices, and natural flavoring agents, either in whole form or as concentrated extracts. Compared to conventional antibiotics as growth promoters (AGP), including penicillin, bacitrasin, tetracyclines, tilmicosin, and tylosin [6], plant-based additives are less toxic, free from harmful residues, and generally more acceptable for long-term use in livestock systems [7] due to their variety of bioactive constituents, including polysaccharides, saponins, polyphenols, and flavonoids, which possess well-documented antioxidants, antimicrobial [8], anti-stress, anti-inflammatory [9], and immune-modulating [10] properties. Notably, these active ingredients are proposed to activate the immune system by stimulating the secretion of digestive juices, enhancing antioxidant activity, and regulating the intestinal flora [11]. Emerging studies have highlighted their potential benefits of *Scutellaria baicalensis* and *L. japonica* extract additives in swine and poultry [12,13,14,15]. Though adding herbal substances to monogastric animals’ diets would be a viable strategy to optimize their production by combating OS, to our knowledge, the existing literature, particularly on Chinese Skullcap (*Scutellaria baicalensis*) and Japanese Honeysuckle (*Lonicera japonica)*, on the health of pigs and poultry remains fragmented. Therefore, this review aims to consolidate current knowledge by highlighting the mechanism of OS, the potential effect of these two herbal extracts, and their effect on monogastric animals.

## 2. Oxidative Stress: Definition and Mechanism

**Figure 1:**
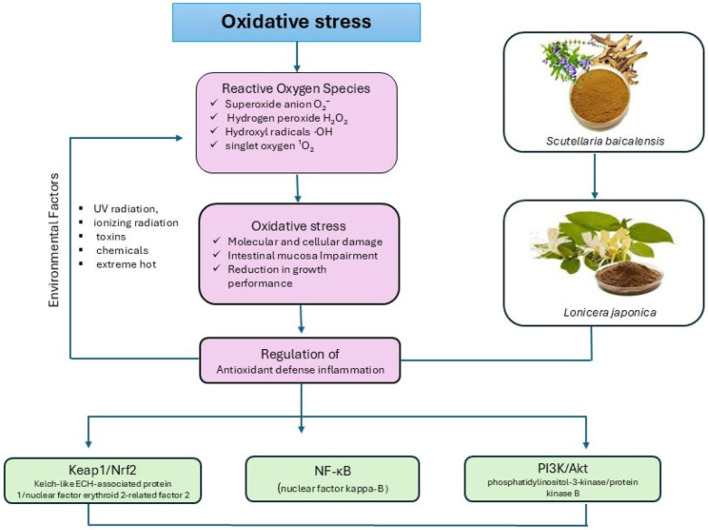
Illustrate the OS definition and mechanism of action.

The term “oxidative stress” refers to a condition in which the physiological balance between pro-oxidants and antioxidants is disrupted in favor of pro-oxidants, leading to molecular and subsequent cellular damage caused by oxidation [16]. Notably, OS leads to the generation of numerous reactive oxygen species (ROS), which are highly reactive molecules formed as intermediates in oxidation processes. It has been reported in the earlier literature that external or environmental factors trigger the generation of ROS, such as ultraviolet radiation, ionizing radiation, toxins, chemicals, and extreme hot environments (Figure 1). Cellular metabolism is accompanied by the production of ROS. These include superoxide anion (O_2_^−^), hydrogen peroxide (H_2_O_2_), hydroxyl radicals (·OH), and singlet oxygen (^1^O_2_) [17]. ROS were unregulated by products of aerobic metabolism and other enzymatic processes that play a critical role in regulating cell function and biological processes. Uncontrolled production of ROS can overwhelm the ability of enzymatic and non-enzymatic antioxidant defense mechanisms, leading to a state of OS and consequently damage biological macromolecules, such as lipids, DNA, and proteins [16]. Also, it causes poor nutrient absorption and digestion, which alters the redox status of the intestinal mucosa and causes the antioxidant system to malfunction. Moreover, OS damages the mucosa of the intestines, impairs the effectiveness of digestion and nutrient absorption, and negatively affects the average growth of animals [18]. Oxidative stress (OS) involves several critical signaling pathways that govern cellular defense, repair, and adaptation to oxidative damage. Among these, the Keap1/Nrf2 pathway stands out as one of the most robust antioxidant defense systems, regulating the expression of protective genes, such as antioxidant enzymes [e.g., superoxide dismutase (SOD), catalase (CAT), glutathione peroxidase (GPX)], detoxifying enzymes, and cytoprotective proteins [19].

## 3. Oxidative Stress: Causes and Influencing Factors in Monogastric Animals

Thermal stress, encompassing both heat and cold challenges, poses significant risks to the health, productivity, and overall welfare of poultry and swine (Table 1). In poultry, heat stress (HS) occurs when birds are exposed to temperatures above their thermoneutral zone (approximately 18–24 °C for broilers), which is particularly detrimental for fast-growing commercial broilers with high metabolic rates [20,21]. Unlike mammals, birds lack sweat glands and cannot effectively dissipate excess heat [22]. Exposure to high temperatures leads to physiological responses, such as elevated body temperature, panting, and altered blood chemistry. Critically, HS induces a redox imbalance that favors the generation of ROS. While the avian body typically maintains redox homeostasis through antioxidant enzymes like GPX and SOD under normal conditions, HS elevates metabolic activity and disrupts mitochondrial function, causing excessive ROS production. This oxidative damage can compromise cell membrane integrity through lipid peroxidation and lead to the oxidation of proteins and nucleic acids, ultimately impairing immune responses, nutrient absorption, growth performance, and meat quality [23]. Similarly, swine are highly susceptible to thermal stress, particularly when exposed to high ambient temperatures (27–33 °C) during gestation or lactation. They often experience OS, reduced antioxidant capacity, and impaired reproductive performance, which adversely affects the growth and survival of their piglets [24]. During late gestation, heat exposure decreases antioxidant enzyme levels and total antioxidant capacity (T-AOC), prolonging parturition and negatively impacting offspring development [25]. In lactating sows, elevated temperatures diminish plasma antioxidant levels and lactation performance, leading to reduced piglet growth. Growing pigs also suffer from heat-induced (35 °C for 1–3 days; 37 °C for 2–6 h) OS, primarily in skeletal muscles, which may be linked to impaired mitophagy and mitochondrial dysfunction [26]. However, research is limited regarding the effects of heat stress on early pregnancy in sows and the neonatal and weaned piglets, particularly in relation to oxidative stress, highlighting a gap in current knowledge.

Cold stress (CS) is a significant environmental stressor that negatively impacts the growth, health, and welfare of both poultry and swine. Young animals in both species are particularly susceptible due to their limited ability to regulate body temperature. In neonatal broiler chickens, immature thermogenic organs hinder effective thermoregulation during early post-hatch development, making them highly vulnerable to cold environments [27]. Similarly, piglets exposed to low ambient temperatures shortly after birth (10–15 °C) often experience impaired absorption of colostral immunoglobulins, increasing the risk of diarrhea and preweaning mortality [28]. At the cellular level, CS significantly alters metabolic processes in both species, leading to increased energy demands and OS. In poultry, cold exposure has been shown to activate adenosine monophosphate-activated protein kinase (AMPK), a critical energy sensor that plays a central role in maintaining energy homeostasis [28]. This activation helps redirect cellular energy to vital processes necessary for stress adaptation [29]. AMPK also plays a protective role in maintaining gut epithelial barrier function, with studies showing that its activation enhances barrier integrity, while its inhibition is associated with dysfunction [30]. Meanwhile, CS can also induce the synthesis of heat shock proteins (HSPs), which serve to stabilize and refold damaged proteins during environmental stress. The expression of HSPs is regulated by heat shock factor 1 (HSF1), a key transcription factor involved in cellular protection [31]. For instance, exposure to temperatures 12 °C below normal in broilers has been associated with inflammatory responses, marked by the upregulation of inducible nitric oxide synthase (iNOS), a key enzyme in inflammatory signaling [32]. Interestingly, in swine, prolonged cold exposure (e.g., 21 days at 15 °C) has been linked to increased total antioxidant capacity (T-AOC) compared to thermoneutral conditions (26 °C), suggesting a degree of physiological adaptation. This may involve enhanced oxidative muscle development and modulation of gut microbiota [24]. However, the specific temperature thresholds that trigger CS responses in sows and weaned piglets remain undefined, underscoring the need for further investigation. As both the poultry and swine industries expand, understanding the shared physiological consequences of CS, including its metabolic, oxidative, and inflammatory effects, is essential for improving animal health, performance, and welfare. Developing effective strategies to mitigate thermal stress will be critical in promoting sustainable and resilient livestock production systems.

Feed toxins are harmful substances present in animal feed, typically produced by microorganisms, such as fungi and bacteria, or introduced through contamination of feed ingredients. The primary sources of feed toxins include mycotoxins, microbial contamination, and chemical pollutants. Fungal toxins, especially those produced by molds such as aflatoxins, vomitoxin (deoxynivalenol, DON), and ochratoxins, are among the most concerning, particularly in poultry and swine production [33]. Mycotoxins in feed pose significant risks not only to animal health and performance but also to human food safety [34]. Bacterial contaminants, such as *Salmonella*, *Campylobacter*, *Clostridium perfringens*, and *Escherichia coli*, are also critical concerns in feed safety research [35,36,37,38]. In addition, chemical contaminants like heavy metals, pesticide residues, and nitrites further exacerbate feed safety challenges. Among these toxins, mycotoxins are known to induce OS by promoting inflammatory responses and cellular damage, while simultaneously inhibiting antioxidant systems in animals. This dual impact amplifies oxidative damage, impairing animal growth and health [39]. In China, contamination of feed with mycotoxins has become a growing concern in recent years. Numerous studies have demonstrated that dietary exposure to DON, one of the most prevalent mycotoxins, can induce oxidative stress and inflammation in pigs, especially in the intestinal tract. For instance, supplementation of 28-day-old weaned piglets’ basal diet with 4 mg/kg DON led to reduced blood catalase (CAT) concentrations. Similarly, exposure to 3.8 mg/kg DON lowered intestinal antioxidant capacity and triggered intestinal inflammation in 21-day-old weaned piglets. Further research has shown that DON exposure at doses ranging from 3 to 12 mg/kg reduces antioxidant capacity in a dose-dependent manner, with the severity influenced by the pig’s age, weight, and developmental stage. Other mycotoxins also exhibit harmful oxidative effects. For example, dietary exposure to 0.25 mg/kg ochratoxin A (OTA) was found to diminish antioxidant capacity in the liver and kidney of piglets, while 0.32 mg/kg aflatoxin B1 (AFB1) reduced antioxidant status in the mesenteric lymph nodes (MLNs), potentially contributing to intestinal barrier dysfunction. These findings highlight the critical need for future research to focus on the OS induced by dietary mycotoxins, particularly in relation to dosage, developmental stage, and physiological condition of the animals.

Lipids in animal feed, particularly those high in polyunsaturated fatty acids (PUFAs), are highly susceptible to oxidation when exposed to high temperatures or improper storage conditions. This oxidation results in the formation of lipid peroxides, such as malondialdehyde (MDA) and 4-hydroxynonenal (4-HNE), which are key indicators of lipid peroxidation and can contribute significantly to oxidative stress (OS) in animals [40]. Proper assessment and control of lipid peroxidation are therefore essential to maintaining feed quality and preventing performance losses, especially in swine and poultry production. In pigs, dietary supplementation with oxidized lipids has been shown to increase oxidative damage. For example, feeding nursery pigs 60 g/kg of oxidized soybean oil (MDA level = 4.5 mmol/L) led to elevated MDA concentrations in the jejunal mucosa, ranging from 35 to 54 μmol/g protein [41]. Similarly, supplementation with 50 g/kg of oxidized fish oil (peroxide level = 186.89 mmol/kg) in newborn piglets significantly raised levels of MDA and oxidized glutathione (GSSG) in the intestinal tissue compared to piglets fed fresh fish oil (peroxide level = 4.20 mmol/kg) [42]. In vitro studies using IPEC-1 cells have further confirmed the peroxidative effects of 4-HNE, demonstrating increased reactive oxygen species (ROS) production and DNA damage, thereby validating its role as a model compound for lipid peroxidation-induced OS in pigs.

In poultry, fats and oils serve not only as concentrated energy sources but also play key roles in enhancing feed digestibility, improving palatability, and supporting the absorption of fat-soluble vitamins (A, D, E, and K) [43]. Additionally, PUFA-rich oils, such as fish and vegetable oils, have been associated with improved immune responses and resistance to pathogens [44]. However, the same PUFAs, due to their multiple conjugated double bonds, are highly unstable and prone to oxidative degradation [45]. When exposed to oxidizing agents, these fats can generate free radicals and lipid peroxides, which contribute to OS and compromise the nutritional value and safety of the feed. Oxidized lipids not only reduce the efficacy of fatty acids but also lead to the formation of harmful compounds, such as aldehydes and carbonyls, which can reduce feed palatability, decrease intake, and impair overall animal health [46]. Research has shown that consumption of oxidized oils in poultry feed can elevate ROS levels and oxidative byproducts, negatively affecting intestinal health and growth performance. For instance, Tavárez et al. [47] reported that yellow-feathered broilers fed oxidized soybean oil exhibited reduced performance due to increased OS. However, some studies, such as those by Anjum et al. [48], suggest that low concentrations of oxidized oil may not significantly impact growth or feed conversion in chicks. Nonetheless, the collective evidence indicates that excessive intake of oxidized dietary fats and oils can disrupt redox balance in both pigs and poultry, highlighting the need for careful management of lipid quality in feed formulations.

**Table 1:** Causes and influencing factors of OS in monogastric animals.

Category	Factor	Species	Mechanism of OS	Consequence	Remarks
Stress	Heat (27–37 °C)	Pig	↑ ROS, ↓ antioxidant enzymes during gestation	impaired reproduction performance and piglet survival	affects late gestation, lactation, and growing stage
		Poultry	↑ ROS from disrupted mitochondria and redox imbalance	↓ immunity, nutrient absorption, meat quality	birds lack sweat glands; fast-growing broilers will be affected mostly
	Cold (10–15 °C)	Pig	↑ T-AOC during prolonged exposure; altered gut microbiota	diarrhea and preweaning mortality	CS threshold in sows/weaned pigs is not well defined
		Poultry	↑ AMPK, ↑ HSPs, and inflammatory enzymes (iNOS)	↓ energy redirection, gut protection, and inflammatory responses	broilers are vulnerable post-hatch due to immature thermogenesis
Feed Toxin	Mycotoxins (e.g., DON, AFB1, OTA)	Pig	↓ antioxidant enzymes Inflammatory signaling	intestinal inflammation, ↓ antioxidant status	DON effect is dose-, age-, and stage-dependent
		Poultry	↓ antioxidant enzymes Inflammatory signaling	↓ performance and immune response	major concern in feed type
	Bacteria (e.g., *E. coli*, *Salmonella*)	Both species	Induces immune responses	Gut inflammation, disease susceptibility	feed safety concern
Lipid Oxidized	PUFA-rich oils (soybean, fish oil)	Pig	MDA, 4-HNE increase → ↑ ROS, DNA damage	Intestinal damage, ↓ growth	nursery pigs and neonates are vulnerable
		Poultry	↑ ROS from PUFA degradation	↓ performance and feed intake	tolerance at low doses

## 4. Characteristics and Biological Activities: *Scutellaria baicalensis* and *Lonicera japonica*

*Scutellaria baicalensis* (*S. baicalensis*, commonly known as Chinese Skullcap) is a perennial plant belonging to the Lamiaceae family, which has a bitter taste and cooling nature, predominantly cultivated in China [49]. Concurrently, it holds a significant place in traditional Chinese medicine for treating liver and lung ailments. The dried root of *S. baicalensis* is especially valued for its rich content of bioactive compounds, including flavonoids, anthraquinones, volatile oils, and other phytochemicals, and plays an important role in livestock production [50]. The characteristic components of *S. baicalensis* are baicalin, baicalein, scutellarin, and scutellarin. Baicalin is a monomer active component extracted from the roots of *S. baicalensis*, while Baicalein is the aglycon form of baicalin [51]. In general, the absorption rate of baicalin is low via oral administration. However, baicalein formed from enzymatic hydrolysis of baicalin in the intestinal tract is easily absorbed into the blood [52]. Flavonoids like baicalin and baicalein are potent antioxidants and operate via two mechanisms. One involves direct hydrogen atom donation, in which phenolic hydroxyl groups lose hydrogen to neutralize ROS. The second mechanism involves a single-electron transfer to stabilize free radicals. The antioxidant function of baicalin is closely tied to its activation of nuclear factor erythroid 2 related factor 2 (Nrf2), a central transcription factor regulating the body’s oxidative stress response. The activation of Nrf2 stimulates the production of antioxidant response elements, helping reduce ROS formation and maintaining cellular homeostasis [53]. Earlier research reported that baicalein is effective in neutralizing various free radicals, including alkyl peroxides and superoxide anions. For instance, Zhao et al. [54] reported that baicalin played an antipyretic role by reducing the concentration of prostaglandin E2 (PGE2) and cyclic adenosine monophosphate (cAMP) in the hypothalamus, while Guo et al. [55] found that reduced heat-stress-induced apoptosis by regulating the Fas/FasL pathway and upregulating heat shock protein 72 (HSP72) expression in bovine testicular Sertoli cells. Some studies exhibit that *S. baicalensis* has strong anti-inflammatory properties and plays a regulatory role in various inflammatory pathways, including cartilage protection [56]. In vitro studies have demonstrated that baicalin mitigates lipopolysaccharide (LPS)-induced inflammation in mammary epithelial cells by suppressing NF-κB activation and p38 MAPK phosphorylation, while also promoting HSP72 expression to reduce inflammation and apoptosis [57]. In animal models, baicalein has been observed to inhibit the production of interleukin-8 and cyclooxygenase-2 (COX-2), while enhancing the expression of HSP70, thus boosting anti-inflammatory defense and preventing inflammation-induced tissue damage [58]. Furthermore, S. baicalensis exerts antiallergic effects mainly by inhibiting the mast cell degranulation process and inhibiting the release of the slow-reacting histamine substance of anaphylaxis (SRS-A). It should be noted that S. baicalensis may alleviate itching, gastrointestinal contraction, and other symptoms caused by type I, II, and IV allergic reactions in animals, without apparent side effects [59].

**Figure 2:**
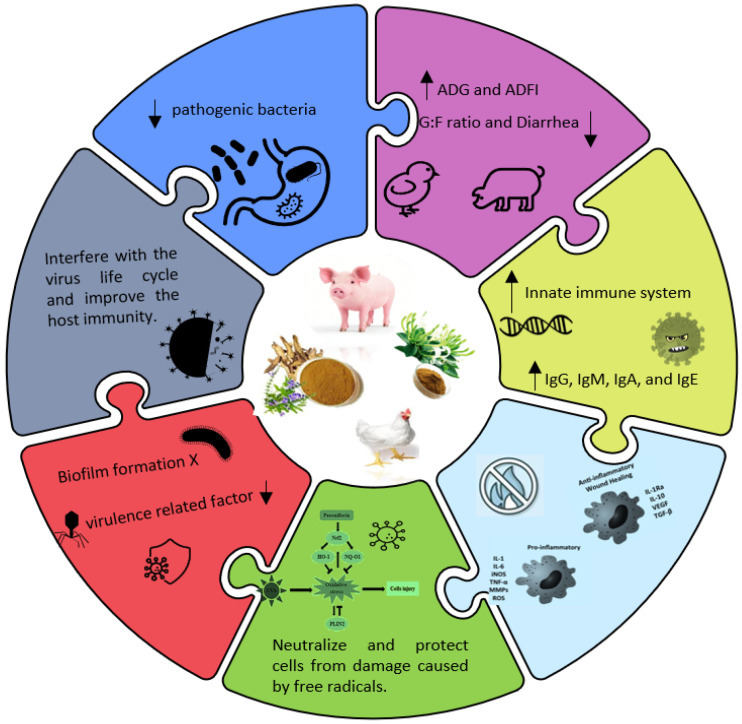
Illustrate the characteristics and biological activities of herbal additives in livestock. Abbreviation: ADG, average daily gain; ADFI, average daily feed intake; G:F ratio, gain to feed ratio; IgG, Immunoglobulin G; IgM; Immunoglobulin G; IgA, Immunoglobulin A; and IgE, Immunoglobulin E; IL-4, Interleukin-4; IL-8, Interleukin-8; TNF-α, tumor necrosis factor alpha; ROS, Reactive oxygen species.

*Lonicera japonica* (*L. japonica*, commonly known as Japanese honeysuckle), a woody, deciduous shrub, belongs to the Caprifoliaceae family. Its flower buds referred to *L. japonicae Flos* have been widely used in traditional Chinese medicine. In addition, the extract of *L. japonica* Thunberg, a homologous herb rich in organic acids, volatile oils, flavonoids, iridoids, and saponins [60,61], is widely used in animal feed due to its diverse pharmacological effects, such as antioxidant, anti-microbial, antiviral, antitoxic, antiseptic, and anti-inflammatory properties [62,63]. Modern pharmacological research has shown that *L. japonica* extract possesses a variety of biological activities, with its antioxidant properties receiving particular attention [64]. These antioxidative effects are largely attributed to their high content of polyphenols [65] and polysaccharides. Previously, Tang et al. [66] found that the antioxidant properties of *L. japonica* positively correlated with the total content of phenolics, flavonoids, chlorogenic acid (CGA), and quercetin. Similarly, Kong et al. [67] observed a strong association between its antioxidant capacity and the levels of CGA, cynaroside, rutin, and hyperoxide. This antioxidant activity is often assessed through well-established assays, such as DPPH and ABTS radical scavenging assays, superoxide radical scavenging activity, ferric-reducing antioxidant power (FRAP), and reducing power (RP) assays [68]. These tests evaluate the ability of the extract to donate hydrogen or electrons and neutralize various free radicals like DPPH, ABTS+, peroxyl, alkoxyl, hydroxyl, and nitric oxide radicals [69]. Polysaccharides, natural polymers formed by glycosidic bonds between aldose or ketose sugars [70], are also key active constituents in *L. japonica*. Several studies have demonstrated that plant-derived polysaccharides can alleviate oxidative stress by exerting strong antioxidant effects [71]. Polysaccharides from *L. japonica* have shown potent DPPH, ABTS+, hydroxyl, and superoxide radical scavenging activities in vitro, along with protective effects against H_2_O_2_-induced erythrocyte hemolysis [72]. In vivo, crude polysaccharides from *L. japonica* have been shown to alleviate oxidative liver damage in streptozotocin (STZ)-induced diabetic rats by reducing serum levels of ALT, AST, and GGT and by enhancing hepatic levels of CAT, SOD, and GSH [5]. These findings emphasize the crucial role of polysaccharides in *L. japonica*’s antioxidant function. Reactive oxygen species (ROS), including radical forms such as O_2_^−^, •OH, and ROO, as well as non-radical species like H_2_O_2_ and O_3_, are continuously produced during cellular respiration and metabolism, primarily in the mitochondria [73]. Under normal physiological conditions, ROS serve important signaling roles in cell growth and adaptation [74]. However, in intensive production systems, animals often suffer from bacterial infections [63], endotoxins [75], mycotoxins [76], and weaning stress [77] that can lead to excessive ROS accumulation, resulting in oxidative stress and cellular damage [78,79]. OS has been implicated in various pathological conditions, including intestinal barrier dysfunction and gastrointestinal diseases [77]. Therefore, reducing oxidative stress is critical for improving animal health and productivity (Figure 2). Although *L. japonica* shows promise in this regard, direct evidence supporting its protective role against intestinal oxidative damage in livestock remains limited and warrants future research. In addition to its antioxidative effects, *L. japonica* has demonstrated significant anti-inflammatory activity in both in vitro and in vivo models [80]. Proinflammatory cytokines, such as TNF-α, IL-1β, and IL-6, are key drivers of inflammation and can initiate a cascade of immune responses [81]. Kang et al. [13] showed that *L. japonica* extract suppressed the release of inflammatory mediators, including IL-6, IL-8, and TNF-α, by inhibiting the NF-κB and MAPK signaling pathways in HMC-1 cells. Likewise, Bang et al. [81] found that BST-104, a water-based extract of *L. japonica*, decreased gastric inflammation by reducing TNF-α, IL-1β, and IL-6 levels in gastric mucosal tissues. These findings strongly support the use of *L. japonica* as a natural anti-inflammatory agent. Moreover, *L. japonica* appears to have beneficial effects on gut microbiota. Yang et al. [82] demonstrated that extracts of *L. japonica* promoted the growth of beneficial bacteria, such as *Lactobacillus*, while inhibiting potential pathogens like *E. coli*, without causing structural damage to the intestine. Considering this, we infer that *L. japonica* could positively influence gut microbiota composition and, consequently, animal intestinal health.

## 5. Effectiveness of *Scutellaria baicalensis* and *Lonicera japonica* in Monogastric Animals

The use of herbal extracts as feed additives in poultry has gained increasing attention due to their reported benefits in enhancing growth performance and health status. These benefits are largely attributed to their antioxidative, anti-inflammatory, and antimicrobial properties [83]. Among the most extensively studied are *Scutellaria baicalensis* and *Lonicera japonica*, both of which have demonstrated a wide range of pharmacological activities in animal studies [83] (Table 2). Zhou et al. [84] reported that dietary supplementation of broilers with 100–200 mg/kg baicalein, a major flavonoid derived from *S. baicalensis*, significantly improved growth performance and immune responses. Specifically, the supplemented broilers exhibited higher CD3+/CD4+ and CD3+/CD8+ T-cell ratios, increased interferon (IFN) levels, enhanced antibody titers, and elevated serum antioxidant enzyme activities, including SOD, GSH-Px, and CAT. Additionally, liver tissues showed increased total antioxidant capacity (T-AOC), total superoxide dismutase (T-SOD), and GSH-Px levels. Further supporting these findings, Króliczewska et al. [85] demonstrated that dietary inclusion of *S. baicalensis* root extract at 1.5% showed no improvement in body weight and feed conversion efficiency in broilers. However, laying hens fed a diet supplementation with 5 g/kg *S. baicalensis* extract not only increased egg weight but also reduced cecal microbial load, lowered propylene glycol content in eggs, and delayed lipid oxidation, indicating potential improvements in egg quality and shelf life [86].

The gastrointestinal tract plays a crucial role in maintaining immune homeostasis and serves as the primary defense barrier against pathogens [87,88,89]. It consists of a complex immune network involving the mucosal layer, epithelial cells, antimicrobial peptides, immunoglobulins, and cytokines [90]. Several studies have highlighted the immunomodulatory role of *L. japonica*, demonstrating its capacity to enhance intestinal immunity and prevent inflammation [25]. The combined use of *S. baicalensis* and *L. japonica* extracts has shown promising synergistic effects. Liu and Kim [91] reported that supplementation of laying hens with 0.25–0.5 g/kg of these extracts mitigated the negative effects of seasonal heat stress, thereby sustaining production performance. Similarly, Kroliczewska et al. [85] observed that broilers receiving this combination exhibited improved growth performance, which was associated with enhanced antioxidant status. At the molecular level, baicalin has been shown to exert anti-inflammatory effects by downregulating TLR4 expression and inhibiting activation of the NF-κB signaling pathway. This mechanism conferred protective effects on the liver of chickens challenged with LPS, thereby reducing inflammation and oxidative stress. However, caution is warranted regarding dosage. Al Amaz et al. [92] observed that it significantly increased the immune status of broilers.

In terms of gut microbiota modulation, baicalin supplementation has been reported to enhance populations of beneficial bacteria, such as *Lactobacillus* and *Bifidobacterium*, while reducing harmful bacteria, including *E. coli* and *Salmonella* [93]. Furthermore, Wang et al. [94] investigated the protective effects of a combined extract of *L. japonica* and *S. baicalensis* (containing chlorogenic acid and baicalin) against *Salmonella pullorum*-induced intestinal damage and dysbiosis. Their findings confirmed that this formulation not only alleviated intestinal injury but also preserved animal performance, primarily through the modulation of gut microbial composition. Also, baicalin supplement showed potent activity against Newcastle disease virus and a direct killing effect against Newcastle disease, capable of inhibiting the infection of chicken embryo fibroblasts and blocking the intracellular Newcastle disease virus and has the potential to be used as a pharmaceutical ingredient [95]. Despite the integration of *S. baicalensis* and *L. japonica* into poultry diets, the formulation shows significant potential for enhancing growth, immune function, and gut health, yet careful consideration of dosage and formulation is essential to maximize benefits while avoiding adverse effects.

Herbal extract mixtures (HEMs) are gaining traction as viable and sustainable alternatives to antibiotic growth promoters in livestock production, largely due to their multifunctional bioactive properties. Among these, *Scutellaria baicalensis* and *Lonicera japonica* have emerged as particularly effective in improving both productivity and health in swine and poultry systems. In swine, numerous studies have underscored the positive impact of HEMs containing these two herbs on animal growth, nutrient utilization, and physiological well-being. For instance, Liu et al. [96] demonstrated that dietary supplementation with a HEM consisting predominantly of *S. baicalensis* (55%) and *L. japonica* (25%) significantly enhanced growth performance and nutrient digestibility in finishing pigs, while also reducing serum cortisol levels, a marker of stress, and improving meat quality. These results indicate that such herbal formulations may offer a dual benefit by supporting both metabolic efficiency and animal welfare. Similarly, Lui et al. [97] found that a complex herbal blend containing *L. japonica* (1000 mg/kg), in combination with *Astragalus membranaceus*, *Eucommia ulmoides*, and *Codonopsis pilosula*, led to improved intestinal structure and increased expression of genes related to nutrient transport, suggesting a direct enhancement of gastrointestinal functionality. These changes are crucial during periods of rapid growth or environmental stress, as they may facilitate more efficient absorption and utilization of dietary nutrients. Fang et al. [98] demonstrated that inclusion of *Scutellaria baicalensis* and *Lonicerae Flos* enhanced the colostrum quality, antioxidant function, liver function, and immunity in sows. Also, they found improved growth performance and immunity in their offspring. Similarly, a Jeong et al. [99] study involving fermented medicinal plant (FMP) mixtures, including *Gynura procumbens*, *Rehmannia glutinosa*, and *S. baicalensis*, confirmed significant improvements in weight gain, nutrient digestibility, and feed efficiency, alongside a notable reduction in harmful gas emissions, such as ammonia and hydrogen sulfide, in broilers. These findings suggest that HEMs may contribute to environmental sustainability in livestock operations. In addition to their performance-enhancing properties, HEMs exert immunological and microbiological benefits. Chang et al. [100] reported that a formulation combining *S. baicalensis*, *Gardenia jasminoides*, and lactic acid bacteria expedited fecal pathogen clearance and modulated gut microbiota enzymatic activity, potentially enhancing the conversion of herbal precursors into biologically active metabolites. Furthermore, *S. baicalensis* at a dose of 1000 mg/kg has been shown to downregulate the expression of inflammatory cytokines by suppressing the NF-κB/p38 MAPK signaling pathway in weaned pigs, highlighting its anti-inflammatory potential at the molecular level.

Reproductive benefits have also been observed. Herbal supplementation during gestation and lactation improved maternal weight retention and litter outcomes in sows [24]. On a cellular level, baicalin, a key compound in *S. baicalensis*, has demonstrated the ability to enhance embryonic development by reducing oxidative damage and apoptosis, optimizing mitochondrial activity, and stimulating the sonic hedgehog signaling pathway. This suggests possible applications of HEMs in reproductive biotechnology and piglet viability. In poultry, *L. japonica* supplementation has shown wide-ranging effects on growth performance, immune status, and product quality. Park et al. [101] reported improved body weight gain, hematological markers, antioxidant capacity, and meat quality in broilers. While Jang et al. [102] did not observe significant changes in meat composition, they found improved antioxidant stability and sensory attributes during cold storage, an important factor in meat preservation and consumer satisfaction. Additionally, chlorogenic acid (GCA), a major phenolic component of *L. japonica*, was effective in promoting broiler growth and reducing infection with *Mycoplasma gallisepticum* when administered via drinking water [103]. In egg-producing hens, *L. japonica*-based supplements have been associated with improvements in laying performance, eggshell integrity, and shelf life of eggs [104]. These findings underscore the potential of *Scutellaria baicalensis* and *Lonicera japonica*, whether used individually or as part of multi-herbal formulations, to act as effective functional feed additives in sustainable livestock production. Their incorporation has been shown to enhance reproductive efficiency, improve gut health, boost immune function, and elevate overall productivity and product quality in both pigs and poultry—contributing not only to commercial success but also to improved food safety and animal welfare.

**Table 2:** Effect of *Scutellaria baicalensis and Lonicera japonica* herbal extracts on monogastric animals.

Animal	Herbal Additive	Dosage	Duration	Observed Effects	Reference
Broiler	*S. baicalensis*	100–200 mg/kg	42 days	↑ growth performance, immune markers (CD3+/CD4+, IFN), antioxidant enzymes (SOD, GSH-Px, CAT), T-AOC in liver	Zhou et al. [84]
Hubbard Hi-Y male broiler	*S. baicalensis* root extract	0.5, 1.0, and 1.5%	42 days	X growth performance, ↑ relative weight of bursa of Fabricius and spleen	Króliczewska et al. [85]
Layers	*S. baicalensis* extract	0–0.5%	14 days	↑ egg weight and egg shelf life ↓ cecal microbes and lipid oxidation	An et al. [86]
ISA brown layers	*S. baicalensis* + *L. japonica*	0, 0.025%, and 0.05%	56 days	↑ Productivity and ↓ serum cortisol concentration	Liu and Kim [91]
Cobb 500	*S. baicalensis* + *L. japonica*	250 mg/kg	35 days	↑ AvBD11, IL4, and TLR21 expression, ↓ TLR15 expression, ↓ IFNg expression under heat stress condition	Al Amaz et al. [92]
Beijing white chickens	*S. baicalensis* + *L. japonica*	0, 50, 100, and 200 mg/kg	35 days	↓ TLR4, ↓ NF-κB activation, ↓ liver inflammation	Cheng et al. [66]
Arbor Acres broiler	*Scutellaria baicalensis* Georgi	0, 60, 120, 180, or 240 mg	42 days	↓ drip loss of thigh muscle ↑ liver T-SOD and GSH-Px activity	Liang et al. [93]
Jinghong laying hens	Flos lonicerae in Combination with Baikal skullcap Attenuate	1000 mg/kg	56 days (Challenged with *S. pullorum* at the end 28 days)	↑ serum endotoxin content, ileal expression of pro-inflammatory cytokines, including IFNG, TNFA, IL8, and IL1B, ↑ Firmicutes, Bacteroidetes and Prevotellaceae	Wang et al. [94]
Ross broilers	Fermented medicinal plants: Gynura, Rehmannia, *S. baicalensis*	0.05% to 0.2%	35 days	↑ body weight gain and feed conversion ratio; ↑ dry matter and nitrogen retention, metabolizable energy, ↓ NH_3_ and H_2_S emissions, ↑ *Lactobacillus* spp. counts and ↓ *E. coli* counts	Jeong and Kim [99]
Ross-308 broiler	*L. japonica*	Challenged	35 days	↑ body weight and immune response in *M. gallisepticum*-infected broiler flocks.	Müştak, H., et al. [103]
Jingfen No. 2 laying hens	honeysuckle extract	100, 200, and 300 mg/kg	35 days	↑ average egg weight and average daily feed intake, ↑ Haugh unit ↓ serum total cholesterol and triglyceride, ↓ yolk cholesterol	Long Bin, et al. [104]
Finishing pig	*Scutellaria baicalensis* and *Lonicera japonica* extract	0, 0.025% and 0.05% herbal extract mixture	84 days	↑ growth, ↑ digestibility, ↓ cortisol, ↑ meat quality	Liu et al. [96]
Sow and offspring	*Scutellaria baicalensis* and Lonicerae Flos	1000 mg per kg feed	From d 80 gestation to d 21 of lactation	↑ colostrum quality, antioxidant function, liver function and immunity in sows, ↑ growth performance and immunity of piglet	Fang et al. [98]

## 6. Conclusions

*Scutellaria baicalensis* and *Lonicera japonica* extracts represent a promising frontier in the development of natural alternatives to antibiotics in animal husbandry. Their rich profiles of bioactive compounds, particularly flavonoids, phenolic acids, and essential oils, offer potent antioxidative, anti-inflammatory, and antimicrobial effects to support animal health and production outcomes. While initial studies highlight their therapeutic potential, especially in maintaining intestinal integrity and immune balance, further in-depth research is essential to fully elucidate their regulatory mechanisms, optimize dosage strategies, and standardize formulations.

## Data Availability

No new data were generated in this study.
